# The spread and antimicrobial resistance of *Staphylococcus aureus* in South African dairy herds – A review

**DOI:** 10.4102/ojvr.v88i1.1937

**Published:** 2021-10-26

**Authors:** Joanne Karzis, Inge-Marie Petzer, Vinny Naidoo, Edward F. Donkin

**Affiliations:** 1 Department of Production Animal Studies, Faculty of Veterinary Science, University of Pretoria, Pretoria, South Africa; 2 Department of Paraclinical Sciences, Faculty of Veterinary Science, University of Pretoria, Pretoria, South Africa; 3 Department of Animal and Wildlife Sciences, Faculty of Agricultural Sciences, University of Pretoria, Pretoria, South Africa

**Keywords:** mastitis, economic loss, dairy, cattle, antibiotic, resistance

## Abstract

*Staphylococcus aureus* is internationally recognised as a principal agent of mastitis and the foremost reason for economic loss in the dairy industry. The limited data available on organism-specific antibiotic resistance surveillance in dairy cattle have stimulated the need for such a review article. The objective of this study was to review relevant literature on antimicrobial resistance of mastitis-causing staphylococci isolated from dairy cows in South Africa compared to other countries. Factors relating to the incidence of mastitis and treatment strategies in terms of the One Health concept and food security were included. The Web of Science (all databases) and relevant websites were used, and articles not written in English were excluded. The incidence of mastitis varied between South Africa and other countries. Antimicrobial resistance patterns caused by *S. aureus* also varied in regions within Southern Africa and those of other countries although some similarities were shown. Antimicrobial resistance differed between *S. aureus* bacteria that were maltose positive and negative (an emerging pathogen). The results highlighted the importance of the availability of organism-specific surveillance data of the incidence of mastitis and antibiotic resistance for specific countries and within similar climatic conditions. Accurate knowledge about whether a specific pathogen is resistant to an antibiotic within a certain climate, country, area or farm should reduce the incidence of unnecessary or incorrect treatment with antibiotics. This should enable dairy farmers to deal with these organisms in a more effective manner. Therefore such research should be ongoing.

## Introduction

Mastitis is a major endemic disease of dairy cattle alongside respiratory disease. It remains the disease in the developed countries that cause most economic losses (Geary et al. 2012). Host-adapted mastitis intramammary infections (IMI) are transmitted from cow to cow. There are several mastitis pathogens for which the primary reservoir is the udder of the cow and the prevalence of IMI because of these pathogens is significant. The coagulase-positive staphylococci are of the most common pathogens in this category, especially *Staphylococcus aureus* (*S. aureus*) (Nickerson, Owens & Boddie 1995).

The udder is the primary reservoir of *S. aureus*, and this organism is transmitted during milking, although a proportion of heifers that enter the milking herd may already be infected with *S. aureus* (Nickerson et al. 1995). This suggests routes of transmission other than milking equipment, hands of milkers’ and udder cloths used during milking. Management and control of *S. aureus* in a dairy herd require insight of the reservoirs of infection and modes of transmission (Rainard et al. 2017). Modern molecular techniques have recently been used to identify different strains of the organism, and these tools are likely to improve the understanding of *S. aureus* epidemiology in dairy herds (Leuenberger et al. 2019).

There is only a small selection of intramammary antibiotics available in South Africa, which are mainly products based on ampicillin and cloxacillin. Antibiotic usage has been shown to correlate with the emergence and maintenance of antibiotic-resistant traits within pathogenic strains in ruminants (Ben Zakour et al. 2008). Dairy cattle producers are particularly affected because of the negative impact resistant strains have on milk production and economics (Bean, Williamson & Cursons 2004). Host-adapted mastitis is controlled with improvements in hygiene and disinfection aimed at disrupting the transmission of the organism from one cow to another, as well as the elimination of infected cows via antibiotic treatment or culling (Barkema, Schukken & Zadoks 2006). The ability to treat mastitis effectively depends not only on the efficacy of the active ingredient of the antibiotic but also on many aspects of management, hygiene, cow immunity, application of the intramammary product and other factors such as pharmacodynamics and udder parenchyma damage. Bacterial susceptibility to an antibiotic, as determined by susceptibility testing, can aid in the appropriate selection of an antibiotic for the treatment of mastitis in dairy cows (Brînda 2009), but this is not necessarily an indication of treatment success. The knowledge regarding whether a specific pathogen is resistant to an antibiotic within a certain climate, country, area or farm should reduce the incidence of unnecessary or incorrect treatment with antibiotics.

The objectives of this study were to review and collate a comprehensive set of literature from South Africa on staphylococci (including *S. aureus*, other coagulase-positive staphylococci and methicillin-resistant [*mecA*] *S. aureus* [MRSA]) causing mastitis in dairy cows and to compare this information to that from other countries. Factors relating to the incidence of mastitis and to treatment strategies in terms of the One Health concept were included. An additional aim was to compare literature on antibiotic resistance of the coagulase positive Staphylococci in South Africa with that reported from other countries.

## Methods

Outline of bibliographic search:

- Full articles from scientific journals, conference papers, proceedings and agricultural reports were considered. When conference papers, reports and proceedings are included, then publication bias is not ruled out.
- Studies that were considered were cohort, cross sectional, case control as well as analytical studies.

Each article was then further studied by obtaining the full text where possible to discover where the study was done and under which conditions that are important to know for mastitis-causing organisms. The time period searched was from 1947 to 2020. Literature from all over the world was included for comparison with South African data. In adherence with the limitation of a number of references permitted for review articles, earlier literature was excluded, when recent literature was available. Articles excluded were those who did not comply with the above criteria, as well as duplicated articles and articles not presented in English. The Web of Science (all databases) was used. These databases were selected for the comprehensive collection of data on agricultural and medical information related to mastitis and antibiotic resistance in dairy cows. The present research was done using search terms singly and then combining the different search terms of the various sub-headings throughout this review for analysis.

## Review findings

### Mastitis

Subclinical mastitis was defined as an infected quarter with a somatic cell count (SCC) ≥ 200 000 cells/mL (Hillerton 1999) or a composite milk samples with SCC ≥ 150 000 cells/mL (Petzer et al. 2017), in the absence of clinical changes to milk. Quarter milk samples with an SCC of ≤100.000 cells/mL milk from which no microorganisms were isolated and without a history of recent infection are considered to be normal (Petzer et al. 2017).

### Causes of mastitis

#### Bacterial species and occurrence

Specific knowledge of mastitogenic pathogens is important to manage the disease. The pathogenicity of an organism to cause mastitis mainly determines the degree of importance. Moreover, the interactions of the environment, the host and agent also are important.

Some studies from Norway, New Zealand, North America, Denmark, England, Ireland, the Netherlands, Germany, Portugal, France, Beijing, South Africa, Ethiopia and Rwanda, between 1975 and 2018, have shown that *S. aureus*, at that time, may have been the most prevalent cause of mastitis with estimates that 7% – 40% of all cows were infected (Bakken 1981; Elliott, Tattersfield & Brookbanks 1975; Fox & Gay 1993; Holmes & Zadocks 2011; Mekonnen et al. 2017; Mpatswenumugabo et al. 2017; Schmidt, Kock & Ehlers 2017; Wang et al. 2018).

Petzer et al. (2009) investigated milk and udder secretions from lactating and dry cows tested for the presence of micro-organisms in a study undertaken from 1996 to 2009 in South Africa. The results from lactating cows (*n* = 379 000) and the dry cows (*n* = 11 946) revealed that non aureus staphylococci (NAS) previously known as coagulase-negative staphylococci (CNS) were by far the most abundant bacteria frequently isolated at 61.71%, followed by *S. aureus* (17.28%) and αβ haemolytic *S. aureus* (STH) (7.81%) and smaller percentages of all other mastitis-causing bacteria. Similar percentages of NAS, *S. aureus*, STH and other organisms were isolated from both lactating and dry cows (Petzer et al. 2009).

In South Africa from 2011 to January 2020, the number of milk producers decreased from 2686 producers to 1164, causing many herds to be amalgamated, therefore with an increased risk for spread of contagious organisms in the absence of adequate biosecurity (Sarrazin et al. 2014; Sayers et al. 2013). The average herd size increased from 167 (2010) to 459 (October 2019) (Milksa 2020). The udder health management system as a whole has improved as these larger herds are mainly of well-managed large commercial dairy herds (Milksa 2020).

**Coagulase-positive staphylococci:** *Staphylococcus aureus*: *Staphylococcus aureus* is the most universal host adapted mastitis pathogen in many dairies, even though the prevalence has decreased because of improved milking hygiene and widely implemented mastitis control strategies (Hillerton 1999). *Staphylococcus aureus* nevertheless has remained a very challenging and important mastitogenic pathogen in South Africa (Petzer at al. 2009, 2012). Petzer et al. (2017) reported a prevalence of 9.05% IMI caused by *S. aureus* in South African dairy herds (2001–2015), whilst a prevalence of 10% – 40% was reported in other countries (Liu et al. 2017). Chronic carrier cows are the major source of infection, shedding the *S. aureus* bacteria intermittently, and this may limit the life and milk production potential of the infected quarter (Mellenberger & Kirk 2001). The design and function of the milking machine and the milking parlour and the resultant milking routine can also predispose cows to mastitis (Dodd & Neave 1970). Flies have also been implicated in the transfer of *S. aureus* from one animal to another (Mellenberger & Kirk 2001).

Udder infection with *S. aureus* often leads to fibrosis, abscesses and recurring clinical mastitis (Mellenberger & Kirk 2001). *Staphylococcus aureus* is especially difficult to manage within a herd, and it can be fatal to the cow (Mellenberger & Kirk 2001). These bacteria have the ability to avoid phagocytosis by producing a polysaccharide mucous biofilm around themselves, leading to poor penetration of the antibiotic during treatment (Parul et al. 2019). In this manner, such bacteria are also further shielded from the defence mechanisms within the cells of the cow (Mellenberger & Kirk 2001).

A study by Monistero et al. (2018) evaluated 120 isolates from four continents and eight different countries: in Europe (Germany and Italy), in North America (New York State and Brazil), in South America (Argentina and Columbia) and in Africa (South Africa and Tunisia). However, only new variants of existing genotypes were detected for five of these seven countries other than South Africa participating in the study, including GTI^V^, GTIV^I^ (Argentina), GTAO^I^, GTAO^II^ (Colombia), GTAQ^I^, GTBN^I^, GTBN^II^, GTBY^I^(Brazil), GTC^V^, GTI^V^ (New York State) and GTR^XIII^ (Italy) (Monistero et al. 2018). New genotypes (GTAR, GTBZ and GTCA) were observed for the South African strains. In contrast to the Colombia, in the Argentinian strain, only one contained the *sak* gene. This showed an apparently clear dissociation from the strains isolated from humans (Monistero et al. 2018). Cosandey et al. (2016) described *S. aureus* genotype B (GTB) only in Italy, whilst *S. aureus* genotype R (CLR) and *S. aureus* genotype C (CLC) were identified in many places in Europe, America and Africa. Genotype CLR, which represents a large cluster containing 13 variants, was detected in all eight countries studied except for Brazil (Monistero et al. 2020). Cosandey et al. (2016) have shown that *S. aureus* CLR and CLC clusters are exclusive to dairy cattle and the spread thereof probably started many years ago, when cattle were moved from Europe to other countries (Monistero et al. 2018). Similarly, the new genotype was found in South Africa by Monistero et al. (2018) who demonstrated the large variety of *S. aureus* genotypes in dairy cattle worldwide. This finding has suggested that monitoring these variations may assist with the reduction of spreading of mastitis organisms, as different genotypes are found and identified in different areas (Monistero et al. 2018).

***αβ* haemolytic *Staphylococcus aureus* lytic group III:** During 1989, a distinct αβ haemolytic *S. aureus* was isolated in South Africa from milk samples collected from a herd with 300 cows in milk (Petzer et al. 2009). These bacteria all belonged to the phage type, lytic group III, whilst all other *S. aureus* isolated from milk from other herds were either from lytic group I or II. A nasal swab was taken from the owner of this herd, and a veterinarian who had chronic sinusitis and *S. aureus* (Lytic group III) (STH) was isolated. Following on this case, numerous other isolates of STH were obtained mainly from milkers over the next 10 years (Petzer et al. 2009). Another South African study of *S. aureus* from dairy herds in Bloemfontein in 1985 had also isolated *S. aureus* (lytic group III) (STH), believed to be of human origin (Swartz, Jooste & Novello 1985).

The percentage of STH isolated from milk of South African cows increased to 20%, which was more than the 13% of *S. aureus* (Lytic groups I and II) isolated in 1999 (Petzer at al. 2009). What is of concern is that the STH seems to be more pathogenic compared to the other *S. aureus* bacteria. Of the bacteria isolated from quarters with mastitis, 67.1% of them were STH isolates, whilst 52.4% of them were from the other *S. aureus* isolates (Petzer et al. 2009). The possible effect of anthroponosis of pathogens from immunosuppressed individuals to cows should not be ignored and warrants further investigation (Petzer et al. 2009; Schmidt, Kock & Ehlers 2015).

**Methicillin-resistant *Staphylococcus aureus* in humans and animals:** Methicillin-resistant *S. aureus* (MRSA) bacteria have an acquired gene that renders them resistant to methicillin and basically to all other beta-lactam antibiotics (Perovic et al. 2006). Methicillin-resistant *S. aureus* is a main source of healthcare-associated, community-associated and livestock-associated infections (Perovic et al. 2006). Methicillin-resistant *S. aureus* is of serious concern in human medicine and an emerging concern in veterinary medicine (García-Álvarez et al. 2011). Milk samples from South African dairy cattle have tested negative for the *mecA* (gene typically indicating methicillin resistance) on the polymerase chain reaction (PCR) but showed phenotypic MRSA using the cefoxitin disc (Badenhorst, Karzis & Petzer 2014). This could have been the strain of *S. aureus* now found to carry a homologue of the *mecA* gene now known as the *mecC* gene (García-Álvarez et al. 2011).

*Staphylococcus aureus* is transmitted amongst humans, amongst animals, from humans to animals and from animals to humans when in close contact (Juhász-Kaszanyitzky et al. 2007). Horizontal transmission amongst humans usually happens via direct contact from hands of infected people or from contaminated food (Perovic et al. 2006).

**Maltose-negative *Staphylococcus aureus:* An emerging udder pathogen:** Staphylococci, which include *S. aureus* and the *Staphylococcus pseudintermedius* and *Staphylococcus intermedius* groups, are responsible for the most clinical diseases in veterinary medicine and are coagulase positive (Sasaki et al. 2007). More recently, based on the nucleotide sequence analysis of the *sodA* and *hsp60* genes, isolates that were maltose negative and previously identified (phenotypic characteristics) as *Staphylococcus intermedius* were reclassified into three clusters, namely: *Staphylococcus intermedius, Staphylococcus pseudintermedius* and *Staphylococcus delphini* (Sasaki et al. 2007).

Whilst there have been studies on multi-drug resistant *S. pseudintermedius* isolated from dogs, cats and horses (Oguttu, Qekwana & Odoi 2017; Van Duijkeren et al. 2011), little work has been undertaken on *S. pseudintermedius* causing mastitis in dairy cattle. Suspected *Staphylococcus pseudintermedius* was initially isolated from one large commercial dairy herd in South Africa in 2005 and 2006, which had been found to have a large number of *S. aureus* with low SCC < 100 000 cells/mL milk (Karzis et al. 2020b). Further identification of these maltose-negative staphylococci using matrix assisted laser desorption ionization-time of flight (MALDI-TOF) mass spectrometry and 16S ribosomal ribonucleic acid (rRNA) found these organisms to be *S. aureus* strain stain type (ST) 2992, an identical strain of single origin, isolated repeatedly over time (Karzis et al. 2020b). Although both the *MalA* and *MalR* genes were present, they were not expressed phenotypically. This may have been attributed to the abnormal stop codon present on the *MalA* gene (Gen Bank accession number: MN531305) (Karzis et al. 2020b). This newly identified maltose-negative *S. aureus* seemed to have low virulence, similar to that of non-*aureus* staphylococci, which is different to that of maltose-positive *S. aureus* (Karzis et al. 2020b).

### Factors affecting the incidence of mastitis in cows: Cow factors

Factors affecting the incidence of mastitis in cows can include anatomical factors such as udder suspension, teat canal, internal blood supply, lymphatic system and previous udder damage, from physical trauma, or damaged parenchyma or previous infections and even including high yields (Sordillo 2009).

#### The immune system of dairy cows

The defence of the mammary gland can be divided into two prominent categories: the innate immunity that is not specific and prevalent in early lactation and acquired or specific immunity, which recognises specific factors of a pathogen (Sordillo 2009).

Cell-mediated immune responses (CMIR) and antibody-mediated immune responses (AMIR) are used as indicators of the adaptive immune responses of livestock (Heriazon et al. 2009). Intramammary infections provoke inflammatory reactions leading to elevated SCC and activate bacteriostatic enzymes in the milk (Bruckmaier & Meyer 2005). Health of dairy cows is influenced by an interaction between the innate components of the immune system and an inflammatory response, which can sometimes be more destructive than helpful (Bertoni, Minuti & Trevisi 2015). Infections, as well as metabolic and parasitic diseases, should, therefore, be avoided as far as possible during the peri-parturient period of dairy cows (Bertoni et al. 2015). The latter can have a negative impact on the dry matter intake, energy efficiency, liver function, fertility and milk yield (Bertoni et al. 2015).

### Antimicrobials

Antimicrobial use has become part of both human and animal living (Lewis 2013). As a result of antimicrobial use in disease control, maintenance of health and applications in the agricultural and environmental sectors, drug resistance has resulted, which now threatens the efficacy of these medicines (Department of Health & Department of Agriculture, Forestry and Fisheries [DAFF] 2018). The first commercially available antibacterial was Prontosil, a sulphonamide, discovered in 1932, introduced in 1936, and resistance was first observed in 1942 (Lewis 2013).

Many people credit Alexander Fleming for developing the first true antibiotic in 1928. This was described as a term invented by Selman Waksman who described it as a compound that was either produced or derived from microorganisms that could kill or hinder the growth of another microorganism (Waksman 1947). Penicillin was discovered in 1928 and introduced in 1938, and resistance was first observed in 1945 (Lewis 2013).

The drugs available for intramammary remedies used in South Africa are mainly limited to cloxacillin and ampicillin combinations in the past, with cephalosporin products introduced into the market more recently. Currently, there are only two dry cow and four lactating cow remedies available for use in South Africa (July 2021).

#### Antibiotics

**Antibiotic classes:** Antibiotics are divided into classes and then further into subclasses. The main antibiotic classes used for the treatment of mastitis are beta lactams, tetracyclines, quinolones, sulphonamides, amphenicols, aminoglycosides, macrolides, lincosamides, polypeptides and pleuromutilins (Gualerzi et al. 2014). There are also many antibiotic subclasses, which are allocated in no consistent manner for some drugs, for example, the beta lactams that are penicillins and cephalosporins. The penicillins are divided by synthesis (natural, biosynthetic and semi-synthetic), penicillinase resistance or duration of action, and the cephalosporins are divided into classes (Gualerzi et al. 2014).

**Antibiotic action:** The difference between bactericidal agents (which kills the organism) and bacteriostatic agents (which inhibits growth temporarily) has been determined under defined laboratory settings and is dependent on the specific agent and bacteria (Pankey & Sabath 2004). For the most effective clinical action, the results from *in vitro* studies need to be combined with relevant pharmacokinetic and pharmacodynamics data to offer a better prediction of efficacy *in vivo* (Pankey & Sabath 2004). For pharmacokinetic information, the ability to maintain relevant plasma and tissue concentrations is important. For pharmacodynamics information, the action of the antimicrobial drugs needs to be determined in relation to time or concentration. The term ‘time dependent’ is indicated by the extent and rate at which microorganisms are killed and will remain unchanged and will not be dependent on the maximum antimicrobial concentration, whilst the minimum inhibitory concentration (MIC) is maintained for a specified time (T > MIC). The term ‘concentration dependent’ indicates the extent to which microorganisms are killed, depending on the antimicrobial concentration in relation to the MIC, for example, fluoroquinolones and aminoglycosides (Cmax: MIC and AU_24_C:MIC) (Van Bambeke & Tulkens 2001).

**Antibiotic spectrum:** The effect of antibiotics can be bactericidal or bacteriostatic for either a small (narrow spectrum) or larger group (broad spectrum) of pathogens (Apua Glossary 2019).

**Intrinsic and acquired resistance:** When bacteria pose inherent resistance to a specific antibiotic, it is able to resist the action of that antibiotic because of its functional characteristics or intrinsic structure. It is important to have knowledge of such intrinsic resistance of pathogens to avoid unsuitable and ineffective therapies (Cox & Wright 2013).

#### Legislation and the use of antibiotics in South Africa

Antibiotics, including intramammary mastitis remedies, are registered in South Africa under two separate Acts, namely: Medicines and Related Substances Act as amended (Act 101 of 1965) for scheduled medicines that are only available on prescription from veterinarians and the *Fertilizers, Farm Feeds, Agricultural Remedies and Stock Remedies Act* (Act 36 of 1947) where antibiotics and stock remedies can be sold over the counter without prescription. There is much evidence to suggest that dairy producers often treat cows with antibiotics symptomatically or without confirmation, and these actions may lead to the ongoing or emerging resistance to antibiotics (Burgos, Ellington & Varela 2005).

**Antibiotic use in animals:** The previous DAFF and the South African Animal Health Association (SAAHA) reported on antimicrobial use in animals. From 2014 to 2015, growth promoters (62%), tetracyclines (17%) and macrolides (11%) were mostly used. The growth promoters used were mainly flavophospholipol (flavomycin), ionophores, zinc bacitracin, olaquindox and tylosin and are antibiotics not used in human health (National Department of Health 2018).

**Antib iotic use in humans:** The use of antibiotics in human medicine in South Africa during 2015 was found to be 21 149 standard units per 1000/population (one standard unit is equivalent to an injection or one tablet). This is a high usage compared to various other countries. The use of broad-spectrum penicillin was 1.3–3.3 times higher than in other BRICS (Brazil, India, Russia and China) countries. However, the use was 0.8 times lower than that in the United Kingdom (UK) or United States (US) (National Department of Health 2018).

#### Antibiotic resistance

The Infectious Diseases Society of America highlighted six groups of pathogens, which are commonly associated with antimicrobial resistance: *Klebsiella pneumoniae, S. aureus, Enterococcus faecalis* and *Enterococcus faecium, Acinetobacter baumannii, Pseudomonas aeruginosa* and *Escherichia coli* (*E. coli*) (ESKAPE) (National Department of Health 2018; Santajit & Indrawattana 2016). Of the ESKAPE pathogens, *S. aureus* is the most common contagious pathogen associated with mastitis (Hillerton 1999), followed by *E. coli* and *Klebsiella pneumonia*. Most of the ESKAPE pathogens are multidrug resistant, and they are primarily responsible for nosocomial infections around the globe (Santajit & Indrawattana 2016).

Antimicrobial resistance information from surveillance data in blood cultures of humans for ESKAPE pathogens in South Africa (National Department of Health 2018) indicated that resistance levels for *S. aureus* had decreased from 36% to 23% over the previous 6 years (National Department of Health 2018). At present, there are limited data available in South Africa on antibiotic resistance surveillance in dairy cattle (Petzer et al. 2012; Schmidt et al. 2015; Van Vuuren, Pickard & Greyling 2007), which has highlighted the need for more detailed information.

At the present time, the main National Centre for Biotechnology Information (NCBI) database, containing the main bacterial genome sequences, has predicted the number of potential resistance genes to be in excess of 20 000 of nearly 400 different types (Davies & Davies 2010). A recent World Health Organization (WHO) report has forewarned that resistance to antibiotics is real and an international threat (WHO 2020), which affects human and animal health care and has an important bearing on the agricultural industry. According to a report by the Food and Drug Association (FDA), approximately 80% of all antibiotics used in the USA went to livestock production (USFDA 2016). Despite recent efforts of many international health organisations (Shryock & Richwine 2010) to control and withdraw antibiotic use in animal husbandry, new antibiotic resistance continues to emerge. In Germany, Denmark and Sweden, consumption of antimicrobial agents used for animals was reported to have decreased in recent years (Koch 2013). Contrary to the above findings, The Netherlands reported that following the ban on growth promoters in 2006, the use of antibiotics for disease treatment increased (Maron, Smith & Nachman 2013). In 2005, the European Union (EU) voted for the total ban of antibiotics as growth promoters in feed animals, whose restriction was effective from 01 January 2006 (EU 2005). Overall, the dairy industry is one of the major contributors to worldwide antibiotic usage and information on the trends of susceptibility and resistance in host adapted pathogens such as *S. aureus* is critical. The European Medicines Agency (EMA) and the European Food Safety Authority (EFSA) have published a joint opinion in which they maintained that the use of antimicrobials in animals needs to be rethought, reduced and replaced (International Dairy Federation 2017). The EFSA has supported the restricted use of antimicrobials in animals for only treatment of infectious diseases.

The South African National Veterinary Surveillance and Monitoring Programme for Resistance to Antimicrobial Drugs (SANVAD) has previously found far lower resistance levels than expected in their surveillance (Van Vuuren et al. 2007). Ten per cent resistance to three antibiotics was found, with lower resistance to mastitis remedies. Nevertheless, 80% of *S. pseudintermedius* that were isolated from infections in dogs and pyoderma were resistant to amoxicillin and 20% were resistant to first-generation cephalosporins. Petzer et al. (2007) found resistance rates of 23% for tetracyclines, 37% for ampicillin and 45% for penicillin in *S. aureus* isolates from milk samples (Henton et al. 2011).

Until 2011, data on antibiotics used in livestock production in South Africa were limited as pharmaceutical companies were not forthcoming with this information and two different acts controlled these medicines (Henton et al. 2011). However, a more recent Ministerial report has been more transparent on the antimicrobials used between 2014 and 2015 and has estimated the use of antibiotics at a ratio of 26% – 74% for animals and humans, respectively. These results varied distinctly from those published from China, India and the USA, where a much higher ratio was indicated for animal use (National Department of Health 2018).

***Staphylococcus aureus* antibiotic resistance in South Africa:** Schmidt et al. (2015) found in a limited study conducted in KwaZulu-Natal that 48% of *S. aureus* isolates were resistant to beta-lactams. A study by Karzis et al. (2019) agreed with this relatively high resistance to penicillin and described a few significant differences in antibiotic resistance between the different seasons and between the provinces of South Africa. It is a concern that with the exception of cefuroxime, for all of the tested antibiotics, in most provinces, the predicted prevalence of resistance was above 50%. The lowest predicted prevalence of resistance for all antibiotics, except for cephalosporins, was in KwaZulu-Natal Province during spring. The least resistance was predicted for cephalosporins in Gauteng Province during winter. The reasons for these differences were obscure (Karzis et al. 2019). A study by Fox et al. (1995) performed at Pullman University (US) also found that season, herd and location played significant roles in the prevalence of IMI and was similar to the study by Karzis et al. (2019). A study by Monistero et al. (2020) that investigated the distribution of antimicrobial resistance genes of *S. aureus* in six countries found that the South African *S. aureus* isolates had the highest complete or partial resistance for spiramycin (100%) and for erythromycin (36.4%) (Monistero et al. 2020). There were 11 isolates analysed of which three (27.3%) were assessed phenotypically to be resistant to ampicillin and penicillin, whilst only one had phenotypic β-lactamase activity initially, but a second isolate showed intermediate resistance to lincomycin. This study by Monistero et al. (2020) tested for the presence of six resistant genes, and *ermB* and *blaZ* were isolated both at a frequency of 36.4% from the South African strains and seven (63.6%) isolates possessed the β-hemolysin (*hlb*) gene. The *sea* and *sak* immune evasion cluster (IEC) genes were present in 90.9% and 100%, respectively, in the South African strains, whilst one strain also contained the sodium channel (*snc*) gene strain.

In line with the virulence, the trends in antimicrobial resistance for the maltose-negative *S. aureus* ST 2992 (Karzis et al. 2020b) also showed a closer similarity with the data for non-aureus Staphylococci than for the maltose-positive *S. aureus* (Karzis et al. 2020a). Unexpectedly, the maltose-negative displayed more resistance (*p* < 0.001) than the maltose-positive *S. aureus* isolates to most of the antimicrobials tested using the MIC method (Karzis et al. 2020a).

***Staphylococcus aureus* resistance in other countries:** The isolates from Argentina and Germany also showed some resistance to erythromycin and lincomycin (Monistero et al. 2020). Amongst the antimicrobial resistance genes that were investigated in the US strains, the *hlb* gene was the most abundant (88.2%), the *sea* gene was present in 52.9% and *blaZ* in 41.2%, whilst the *erm* genes and the *sak* and *scn* were not detected (Monistero et al. 2020).

From the Italian isolates, 10 were indicated with penicillin resistance, nine (52.9%) were resistant to ampicillin and in the nine isolates, phenotypic β-lactamase activity was identified (Monistero et al. 2020). Only one isolate from Argentina was phenotypically assessed as resistant to both ampicillin and penicillin, whilst two (12.5%) were β-lactamase positive based on the nitrocefin method (Monistero et al. 2020).

One isolate from Germany was resistant to penicillin, oxacillin and ampicillin, and three were resistant to cephalosporins (cefoperazone, cefazolin and cefquinome). Two other isolates from Germany were resistant to sulfamethoxazole/trimethoprim and one had intermediate resistance to cefoperazone (Monistero et al. 2020).

A study performed in Tennessee found 82 (34.3%) (*n* = 239) *S. aureus* isolates, with resistance to at least one of the 10 antimicrobials tested (Abdi et al. 2018). There were dominating clonal patterns amongst isolates, with resistant isolates belonging to two main Pulse field gel electrophoresis (PFGE) types (Abdi et al. 2018). The prevalence of antibiotic resistance was mostly for tetracycline and varied within and between farms over time (Abdi et al. 2018). In a study performed in Ethiopia, except for penicillin, tetracycline and sulphamethoxazole/trimethoprim, resistance to other drugs was rare (Kalayu et al. 2020).

***Staphylococcus aureus* resistance in South Africa compared to that of other countries:** The resistance of the maltose-positive *S. aureus* observed in a South African study (Karzis et al. 2020a) was in agreement with that reported in other studies in Denmark and New Zealand (Salmon et al. 1998). The relatively high resistance rates of *S. aureus* to penicillin and tetracycline in both the South African studies (Karzis et al. 2020a; Schmidt et al. 2015) also corresponded with the findings of a recent study in Ethiopia (Kalayu et al. 2020). Similarly, as in South Africa, most isolates from the United States (New York State), Argentina and Germany showed resistance to spiramycin (Monistero et al. 2020). In contrast to isolates from South Africa, Ethiopia and the United States of America, spiramycin and/or penicillin were not effective in 58% of Italian isolates (Monistero et al. 2020). Although the Ethiopian study did not test for spiramycin resistance, very high resistance to penicillin was shown (Kalayu et al. 2020). Studies in both animal and human medicine identified pan and multidrug resistance in *S. aureus* isolates in other countries (Haran et al. 2011; Karzis et al. 2020a).

#### The importance of antimicrobial resistance in One Health approach

The One Health initiative encourages interdisciplinary collaboration amongst the human, animal and environmental sectors globally. It is based on the view that such an interdependence will advance future health care and impact positively on longevity and quality of life (Essack 2018).

Thus, a One Health approach is required in order to understand and control antimicrobial resistance (WHO 2020).

According to Essack (2018), bacterial resistance to multiple antibiotic classes is increasing, and strains are exhibiting multiple resistant mechanisms. Resistance genes as well as virulence factors are carried on different portable genetic elements, which are capable of exchange amongst and between bacteria in animals, humans and the environment (Essack 2018).

### Implications and recommendations

This study summarises the latest and most relevant data available on mastitis causing staphylococci in South Africa and abroad on the emergence, spread and antibiotic resistance. This review concentrates mainly on coagulase-positive staphylococci. The most recent South African studies have been reported in the context of the wider field of mastitis and antibiotic resistance worldwide and also in terms of the One Health Concept, which links the animal, human and environmental components of the literature.

One of the key outcomes of this study has shown the importance of the availability of antibiotic resistance surveillance data, specifically for *S. aureus* and other mastitis causing staphylococci. In particular, such pathogens would be the traditional maltose-positive *S. aureus* and the emerging pathogen maltose-negative *S. aureus*. The accurate identification of antibiotic resistance trends and correct treatment should also be likely to reduce the risk of the development of multi-drug-resistant organisms. Such information about the antibiotic resistance trends should enable dairy farmers to deal with these organisms in a more effective manner in the different regions of southern Africa and worldwide in different countries and under various climatic conditions, without having to perform extensive testing each time.

The availability of antibiotic resistance surveillance data for *S. aureus* in dairy cattle is essential in the context of general public health, by facilitating a safe dairy industry, controlling the incidence of antibiotic resistance in dairy cows and contributing to food security. This might include the need to take account of the possibility of zoonosis (transmission from animals to humans) and anthroponosis (transmission from humans to animals) of antibiotic-resistant bacteria.

Mastitis and antibiotic treatment have great financial implications for the milk producer when assessing the cost of treatment and also the cost incurred in discarding the milk for the duration of the withdrawal period.

Thus, more information on the effective and prudent use of antibiotics and the effective management of mastitogenic pathogens by intramammary treatments should reduce expenses for the producers and improve their profitability and sustainability. The development and implementation of a National Antimicrobial Resistance Strategy Framework (NARSF) are main steps towards containing the threat of antimicrobial resistance in animal and human health in South Africa (DAFF 2018). The responsibility for reducing antibiotic resistance requires a collective effort, and therefore global partnerships need to be strengthened. One of the South African research studies showed the importance of good udder health management, which led to a reduced incidence of antimicrobial resistance of *S. aureus* infections in 20 well-managed herds studied over an 11-year period (Isolates: *n* = 5942). This programme was the main factor resulting in the difference in the management of antibiotic resistance between South Africa and other countries.

In subsequent studies in South Africa and in other countries, research has implied that additional factors such as environmental factors, seasonal and regional differences also had an effect on antimicrobial resistance and the efficacy of antimicrobial drugs. However, there are many more factors, which need to be considered in future research. Thus, the existing research shows the importance of the collaboration of the animal-human-environmental interfaces of the One Health concept.

This review has shown not only some similarities but also many variations in antibiotic resistance patterns of *S. aureus* in regions of Southern Africa and other countries. This highlights the importance of organism specific information in each specific region and under similar climatic conditions, in order for such information to be able to be applied in practice. This is why the existence of organism-specific antibiotic surveillance data in all countries is so important and should be continued as ongoing studies and part of the One Health Concept.

The differences in antimicrobial resistance patterns between the maltose-positive and -negative *S. aureus* and other staphylococci have been emphasised. This is valuable information for both veterinarians and producers, in order to adapt management procedures, and for development of prudent treatment protocols to manage the disease in an effective manner.

This review has focussed on the importance of individual bacterial strains in antimicrobial resistance. The antimicrobial resistance of maltose-negative *S. aureus* ST 2992 was higher than that of maltose-positive *S. aureus*, and it was more multi-drug resistant.

Future research is necessary on the incidence of antibiotic resistance in dairy herds in South Africa and to identify and evaluate other mastitis causing pathogens, such as non-aureus staphylococci, *Streptococcus Uberis, Streptococcus Agalactiae, Streptococcus dysgalactiae* and Gram-negative bacteria.

## Conclusion

Mastitis is the disease in dairy cows that is responsible for the greatest economic loss. Its incidence is influenced by the individual cow, the micro-organisms, the environment and management. Mastitis caused by *S. aureus* is still an important problem in udder health in South African dairy herds. This review has shown not only some similarities but also a great variation in antibiotic resistance of *S. aureus* between different regions of Southern Africa and other countries. A difference of antibiotic resistance was also shown between maltose-positive and maltose-negative *S. aureus* (emerging pathogen) and between different levels of management. All this information highlights the importance of good udder health management and organism-specific antibiotic resistance data, for each specific country or situation and under similar climatic conditions. This is essential in order to be able to apply the appropriate management correctly. In future, it is vital that such studies should be continued in an ongoing programme, in order to be able to add to and improve the current surveillance data available for *S. aureus* in dairy cattle worldwide.

In South Africa, there have been only limited data available on antibiotic resistance surveillance in dairy cattle, which has stimulated the need for more detailed information to be generated through appropriate research. This knowledge will be valuable to veterinarians and producers when using antibiotics and for the treatment of dairy cattle in different regions and to assist in developing the policy on access and use of antibiotics. Resistance to antibiotics is the biggest threat to public health and has the ability to impact society negatively. However, the focus worldwide and in South Africa is now on prudent use of antibiotics, which should have an improved effectiveness, reducing the likelihood of any consequent resistance.
